# Studies on the Heterogeneity and Metabolic Activity of Histones from Rabbit Bone Marrow Cells

**DOI:** 10.1038/bjc.1963.52

**Published:** 1963-06

**Authors:** E. J. Hidvégi, I. Árky, F. Antoni, V. Várterész


### STUDIES ON THE HETEROGENEITY AND METABOLIC ACTIVITY OF HISTONES FROM RABBIT BONE MARROW CELLS

E. J. HIDVÉGI, I. ÁRKY, F. ANTONI AND V. VÁRTERÉSZ

*From the Institute for Radiobiological Research, Budapest XXII, Hungary*

Received for publication March 15, 1963

In the last years there have been many studies on the heterogeneity of histones (Davis and Busch, 1959 ; Luck, Rasmussen, Satake and Tsvetikov, 1958 ; Johns, Phillips, Simson and Butler, 1960 ; Hnilica, Johns and Butler, 1962) and these may throw light on the structure of deoxyribo-nucleoproteins (Phillips and Simson, 1962), and on the heterogeneity of DNA.

Busch and his collaborators (Davis and Busch, 1959 ; Davis and Busch, 1960 ; Byvoet and Busch, 1961) have reported the presence in the nucleoprotein from malignant tissues of histone, which is completely absent from rapidly dividing normal tissues such as rat embryo or regenerating liver. A fast rate of incorporation of [$^{14}$C]lysine was characteristic of the tumour-specific histone fraction (RP2-L).

This paper presents some observations connected with the heterogeneity of histone from rabbit bone marrow cells.

### MATERIALS AND METHODS

The bone marrow cells were obtained from normal rabbits (1·5–2·0 kg. in weight). The cells were suspended in cold Hanks' solution, filtered through a stainless steel filter, centrifuged and suspended again in Hanks' solution fortified in glucose (1 mg./ml.) and $NaHCO_3$ (2 mg./ml.).

A suspension containing $10^8$ cells per ml. was incubated at 37° C. in 5 $\mu$c/10 ml. of [U-$^{14}$C]lysine monohydrochloride (Radiochemical Centre) for 20, 60 and 180 minutes. 10 ml. aliquots of the suspension were diluted with 5 volumes of cold Hanks' solution and centrifuged immediately. The sedimented cells were washed 3–4 times with Hanks' solution and suspended in 5 volumes of 40 per cent cold glycerol containing 0·14 M NaCl and 1 mM $MgCl_2$. Nuclei were prepared in glycerol (Antoni, Hidvégi and Lónai, 1962 ; Hidvégi, Lónai and Antoni, 1963) then washed repeatedly with 0·14 M NaCl.

The histone was extracted by homogenization with 10 ml. of 0·25 N HCl in a glass homogenizer, stirred for 30 minutes at 0° C. and finally centrifuged at 3000 r.p.m. The extraction was repeated and the combined extracts centrifuged for 30 minutes at 100,000 × g. (Spinco L. model). The supernatant was dialysed against 0·5 N acetic acid at 4° C. The protein content, the optical densities at 260 and 280 m$\mu$ and the radioactivity of the dialysates were determined.

The histones were chromatographed on a carboxy-methyl-cellulose column (1 × 15 cm., Serva, Heidelberg, capacity : 0·72 m-equiv./g.) according to the method of Davis and Busch (1959). The volume of the mixer was 140 ml. the reservoir contained 1 N formic acid which was changed to 8 N formic acid after the elution of 80 fractions (3 ml.), when a further 40 fractions were eluted. The flow-rate was 15 minutes/fraction.

On the basis of the chromatographic patterns of both radioactivity and protein concentration of histones, 2–4 tubes of each histone peak were pooled in order to assay the extinction at 280 and 260 m$\mu$ and the pH. The radioactivity was measured in an aliquot part by methane gas flow counter (type Friesecke-Hoepfner) and the specific activity of each histone fraction expressed as counts/min./ml./$E_{280}$.

Fig. 1.—Chromatographic patterns of both radioactivity and acid-soluble nuclear proteins of rabbit bone marrow cells, incubated with [$^{14}$C]lysine for 1 and 3 hours, respectively, *in vitro*. The data represent averages of duplicate experiments. Extinction was measured at 280m$\mu$ in each effluent fraction (3 ml.); radioactivity in 0·5 ml. aliquots.

### RESULTS AND DISCUSSION

The chromatographic patterns of the histones of bone marrow incubated for 60 and 180 minutes with [$^{14}$C]lysine *in vitro* are shown in Fig. 1 and consist of 9–10 protein peaks and are similar to those of spleen (Davis and Busch, 1959).

The distribution of radioactivity in the fractions seems to be quite different from the ones found in a variety of organs by Busch and his collaborators (Davis and Busch, 1959, 1960) who found the [$^{14}$C]lysine incorporated into not more than three fractions while in marrow it is incorporated into all of the fractions. The extent of incorporation increases with time.

TABLE I.—*Specific Activity, Ratio of E 280/260 and pH of the Various Histone Peaks Eluted from the Column*
*Each Histone Peak Represents a Pool of 2–4 Tubes after Chromatography*

| Chromatographic peak | Specific activities* after 1 hour† | Specific activities* after 3 hours† | E 280/260 | pH |
|---|---|---|---|---|
| A/a | 13·6 | 32·8 | 1·05 | 2·57 |
| A/b | 24·2 | 39·4 | 1·12 | 2·42 |
| B/a | 21·4 | 27·8 | 1·13 | 2·34 |
| B/b | 25·2 | 49·8 | 1·33 | 2·28 |
| B/c | 35·2 | 88·6 | 1·12 | 2·23 |
| C/a | 26·4 | 49·6 | 1·13 | 2·16 |
| C/b | 25·6 | 42·4 | 1·12 | 2·10 |
| D | 19·2 | 30·2 | 1·02 | 2·05 |
| E | 82·2 | 125·4 | 1·15 | 1·96 |
| F | 19·3 | 26·0 | 1·05 | 1·75 |

* Specific activities are expressed as counts/min./ml./$E_{280}$.
† Averages of duplicate experiments.

As it appears from Table I high specific activities were found in fraction B/c and E. The heavily labelled fraction E is found in all normal tissues but B/c is usually absent (Davis and Busch, 1959; Davis and Busch, 1960). Though B/c represents only a small part of the total histone of bone marrow, on the basis of the high specific activity and according to the pH of fractions from the chromatographic column, it seems to be identical with the fraction RP2-L of histone considered to be characteristic of tumours by Busch and his associates (Davis and Busch, 1959; Davis and Busch, 1960).

The similarity of the histone patterns from rabbit bone marrow and rat spleen (Davis and Busch, 1959) is probably due to the fact that they are both blood-forming organs.

The fact that in our experiments the RP2-L fraction was found in bone marrow may suggest that a characteristic histone exclusive to malignant tissues does not exist. On the other hand, Busch and his colleagues (Davis and Busch, 1959, 1960), who examined all important organs except the bone marrow, found RP2-L only in tumours and this was confirmed by one of the authors (E.J.H.) in the Lettré's laboratory for Walker carcinosarcoma and Lettré-Ehrlich ascites tumour cells (Ballweg, 1961).

In our laboratories we were able to demonstrate the RP2-L histone fraction in lymphoid organs of leukaemic mice, but not in those of animals without leukaemia. Therefore the possibility has to be considered that the bone marrow, because of its pluripotency, behaves anomalously.

The presence of the RP2-L fraction in the normal bone marrow suggests that this histone protein found characteristically in malignancy of bone marrow, leukaemia, does not seem to be a new protein, but only that its quantity (and in part its turnover) increases. Presumably, the appearance of a greater quantity of RP2-L in the course of malignant change is related to the change of metabolic control.

The authors wish to express their thanks to Professors P. C. Koller, P. Alexander, J. A. V. Butler and Dr. D. M. P. Phillips for their helpful advice and criticisms of our experiments or the reading of the manuscript.

SUMMARY

The metabolic activity and the heterogeneity of histone proteins obtained from rabbit bone marrow cells were studied *in vitro*. The histone proteins proved to be extremely heterogeneous resembling those of the spleen. On the basis of chromatographic study and [$^{14}$C]lysine metabolic activity one of the fractions was found to be similar to the RP2-L accepted as characteristic of tumorous tissues. The possible role of this fraction in the development of leukaemia is discussed.

### REFERENCES

Antoni, F., Hidvégi, E. J. and Lónai, P.—(1962) *Acta physiol. hung.*, **21**, 325.
Ballweg, H.—(1961) Lecture at The 5th Hungarian Oncological Congress, Budapest.
Byvoet, P. and Busch, H.—(1961) *Nature, Lond.*, **192**, 871.
Davis, J. R. and Busch, H.—(1959) *Cancer Res.*, **19**, 1157.—(1960) *Ibid.*, **20**, 1208.
Hidvégi, E. J., Lónai, P. and Antoni, F.—(1963) *Z. Krebsforsch.* **65,** 471.
Hnilica, L., Johns, E. W. and Butler, J. A. V.—(1962) *Biochem. J.*, **82**, 123.
Johns, E. W., Phillips, D. M. P., Simson, P. and Butler, J. A. V.—(1960) *Ibid.*, **77**, 631.
Luck, J. M., Rasmussen, P., Satake, K. and Tsvetikov, A. N.—(1958) *J. biol. Chem.*, **233**, 1407.
Phillips, D. M. P. and Simson, P.—(1962) *Biochem. J.*, **82**, 236.


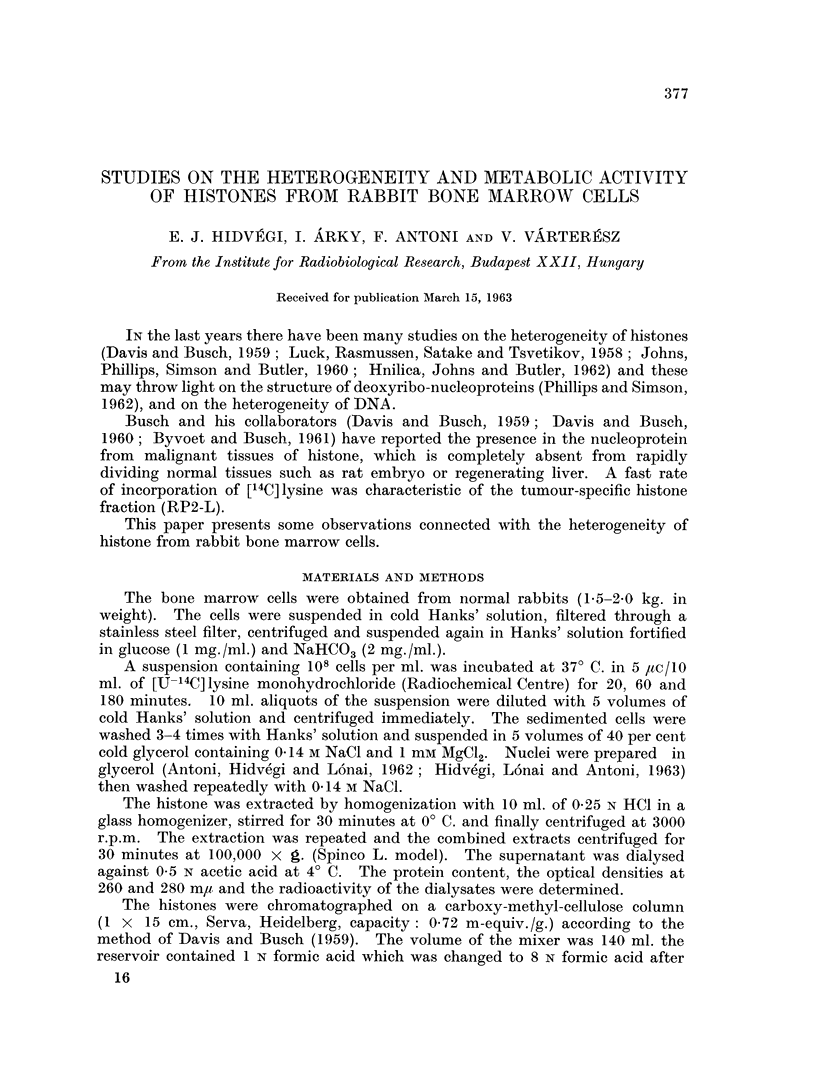

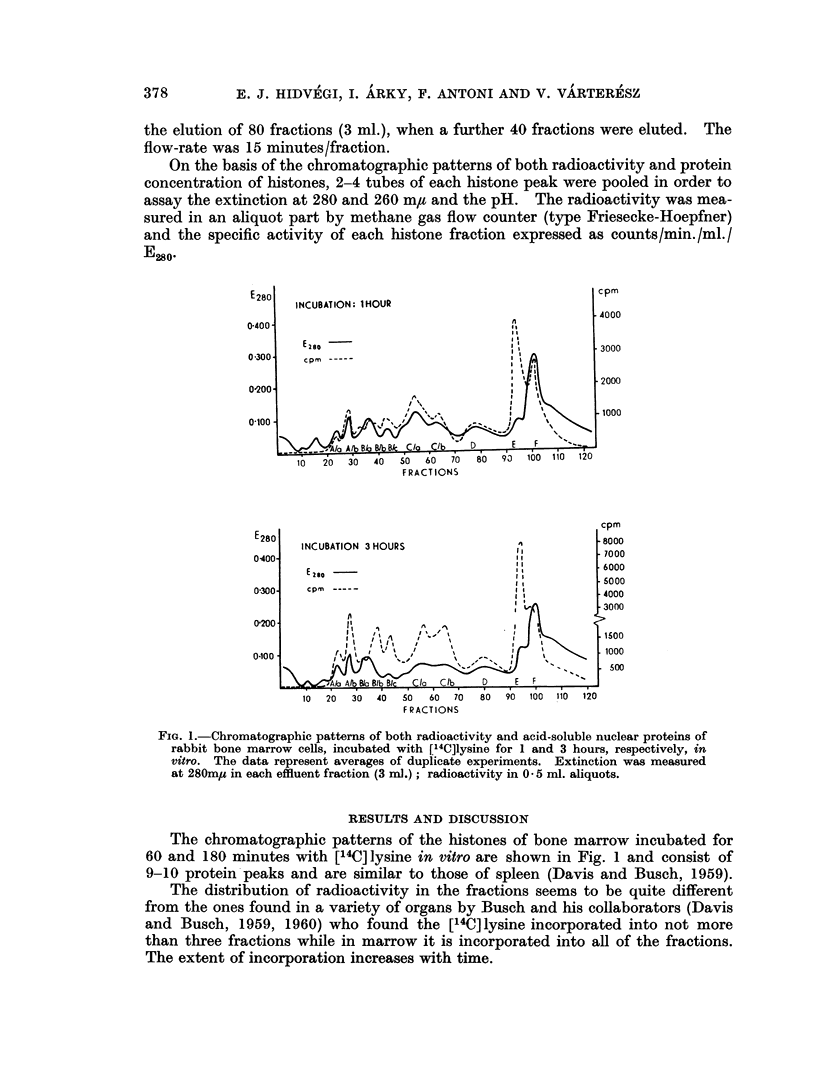

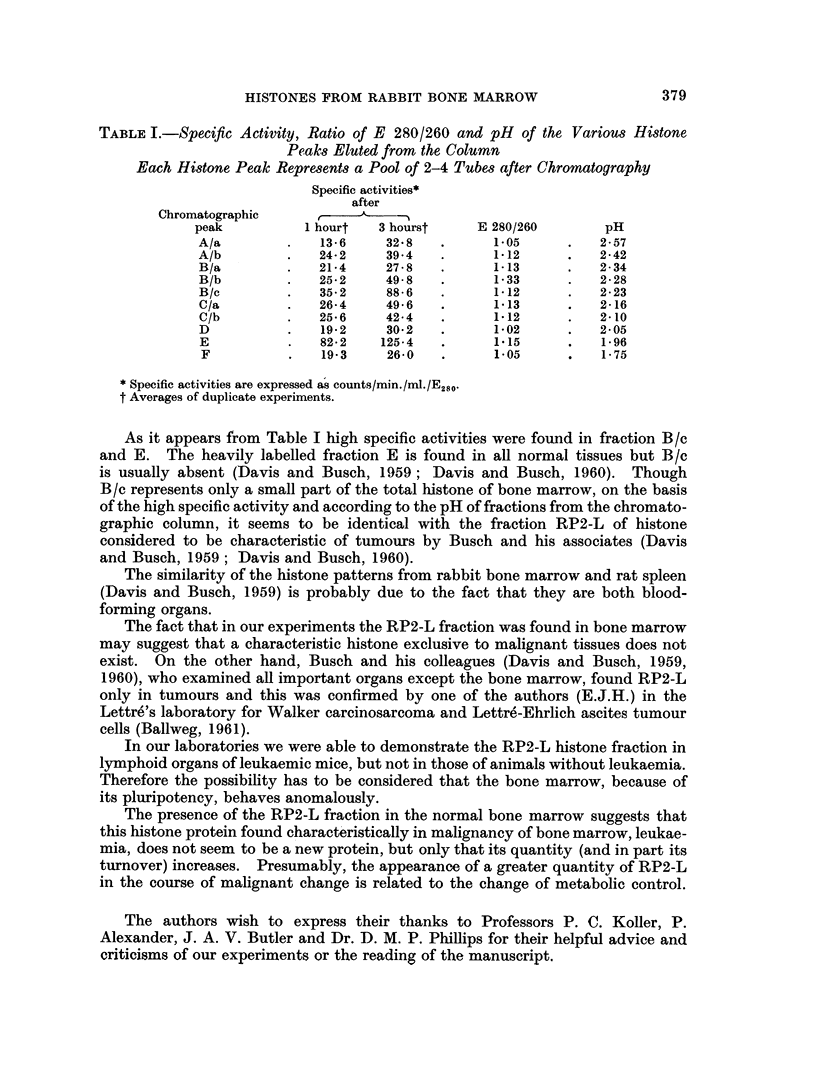

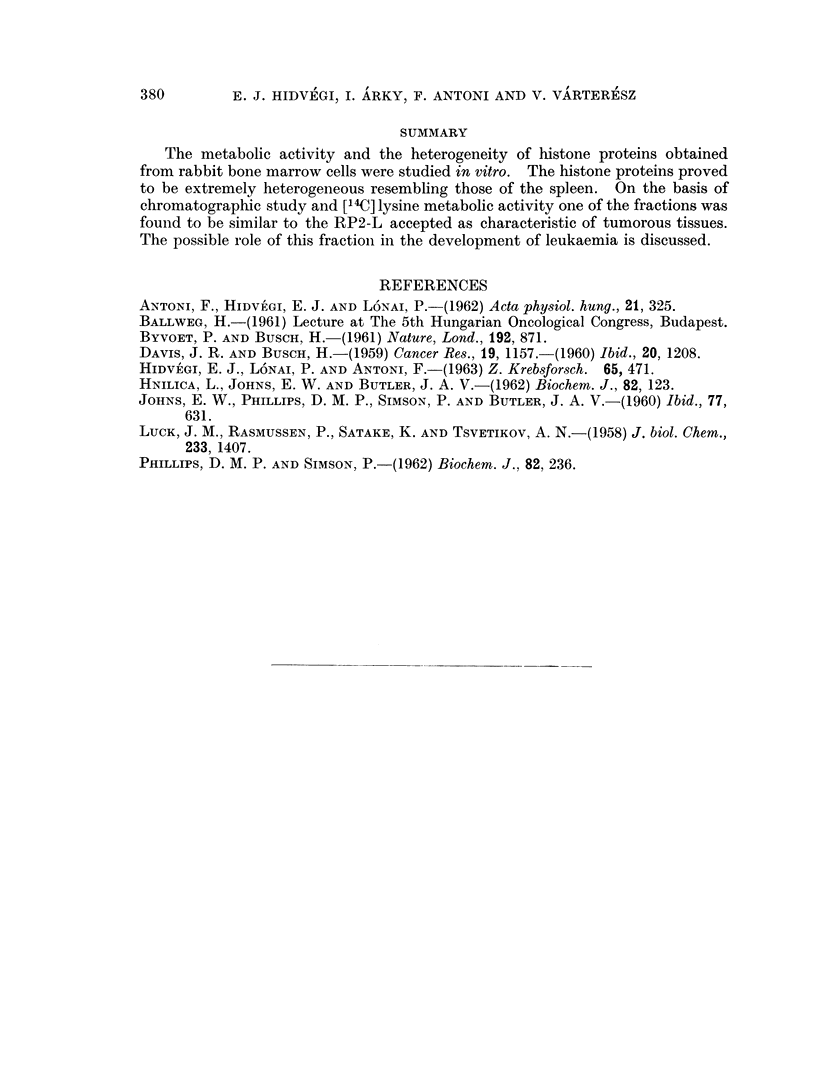


## References

[OCR_00211] ANTONI F., HIDVEGI E. J., LONAI P. (1962). Isolation of cell nuclei from Lettre-Ehrlich ascites tumour cells in glycerol medium.. Acta Physiol Acad Sci Hung.

[OCR_00214] BYVOET P., BUSCH H. (1961). Binding of RP2-L--a nuclear protein of neoplastic tissues--to deoxyribonucleic acid.. Nature.

[OCR_00216] DAVIS J. R., BUSCH H. (1960). Chromatographic analysis of cationic nuclear proteins of a number of neoplastic tissues.. Cancer Res.

[OCR_00217] HIDVEGI E. J., LONAI P., ANTONI F. (1963). [Isolation of ascites tumor cell nuclei in glycerin medium].. Z Krebsforsch.

[OCR_00219] HNILICA L., JOHNS E. W., BUTLER J. A. (1962). Observations on the species and tissue specificity of histones.. Biochem J.

[OCR_00225] LUCK J. M., RASMUSSEN P. S., SATAKE K., TSVETIKOV A. N. (1958). Further studies on the fractionation of calf thymus histone.. J Biol Chem.

[OCR_00229] PHILLIPS D. M., SIMSON P. (1962). Identification of some peptides from an arginine-rich histone and their bearing on the structure of deoxyribonucleohistone.. Biochem J.

